# Concurrence of Rheumatoid Arthritis and Ankylosing Spondylitis: Analysis of Seven Cases and Literature Review

**DOI:** 10.1155/2022/8500567

**Published:** 2022-05-28

**Authors:** Bryan-Josué Flores-Robles, Eztizen Labrador-Sánchez, Estíbaliz Andrés-Trasahedo, Valvanera Pinillos-Aransay, María-Yasmina Joven-Zapata, Laura Torrecilla Lerena, Osman-Alberto Salazar-Asencio, Juan-Antonio López-Martín

**Affiliations:** ^1^San Pedro Hospital, Logroño, La Rioja, Spain Rheumatology Department; ^2^San Pedro Hospital, Logroño, La Rioja, Spain Nursing Department; ^3^Son Espases University Hospital, Palma, Islas Baleares, Spain Neurosurgery Department, Spain

## Abstract

**Introduction:**

The association of rheumatoid arthritis (RA) and ankylosing spondylitis (AS) in a single patient is a rarely described phenomenon. AS and RA are conditions that can have a high impact on the morbidity and mortality of patients.

**Methods:**

We described the clinical, epidemiological, analytical, and radiological characteristics of 81 patients with concomitant diagnosis of rheumatoid arthritis (RA) and ankylosing spondylitis (AS). Of these patients, seven were diagnosed at our hospital. A literature review was carried out using Medline, Embase, Scopus, and virtual hospital libraries, including the period from January 1950 to April 2020.

**Results:**

Regarding the results, 71% of the patients were men, with a mean age of 53 years (±14.83). RA was the first disease diagnosed in 52% of the cases. Approximately 53% of the patients had rheumatoid nodules, and 83% reported inflammatory lumbar pain during their evaluation. Erosions were observed on radiographs of the hands and/or feet in 85% of the cases, and almost all the patients (80/81) had sacroiliitis on imaging studies. Approximately 92% of the cases were rheumatoid factor (RF) positive and 90% HLA B-27 positive.

**Conclusions:**

The coexistence of RA and AS is highly uncommon. With the data obtained in this review, it seems that there exist erosive radiological patterns, positivity for RF, involvement of the axial skeleton, and rheumatoid nodules at a higher frequency than those patients with a single diagnosis of the two entities. More data are needed to corroborate this association.

## 1. Introduction

The association of rheumatoid arthritis (RA) and ankylosing spondylitis (AS) in a single patient is a rarely described phenomenon [1]. AS and RA are conditions that can have a high impact on both the quality of life and the morbidity and mortality of patients [2–4]. Initially, both diseases can manifest similar clinical symptoms such as morning stiffness, pain of an inflammatory nature, and arthritis [2–4]. For this reason, in a patient with RA and axial affectations or a patient with AS and symmetric peripheral joint affectations, it is advisable to extend the study to determine if there is a concurrence of these two diseases [4]. Until the 1970s, both processes were not fully distinguishable from one another; however, the discovery of HLA B-27 and its association with AS marked a turning point.

RA is a disease that predominantly affects women, with a prevalence of 0.5–1% and a typical clinical presentation consisting of symmetric polyarthritis and small joints, with the axial skeleton usually remaining unchanged [1]. In contrast, AS is a disease that predominantly affects male, with a prevalence of 0.1–0.2%, whose main clinical manifestation is inflammatory axial pain and which can manifest with peripheral arthritis in up to 30% of cases (mainly asymmetric and in the lower limbs) [3]. The exact incidence of the coexistence of both diseases in the same person is unknown. However, according to published data, it is estimated that it oscillates between 1 in 500,000 and 1 in 2,000,000 [3, 4]. Up until 1995, approximately 44 cases [3, 4] had been recorded, with a number of discrepancies existing in the literature review; however, the research here presented describes 81 cases (including 7 from our own center).

## 2. Methods

The RA database of our center (658 patients) was reviewed beginning with the first diagnosis of the association between RA and AS. A total of 18 HLA B-27+ patients were selected. Of these, 7 met AS classification criteria [5] and, as a result, were evaluated by the same physician in person on a single occasion, after having signed the informed consent form.

Subsequently, a literature review was carried out (including the period from January 1950 to April 2020) using the following terms: rheumatoid arthritis, ankylosing spondylitis, coexistence and/or concomitance, and/or association and/or concurrence. A total of 42 articles were obtained, of which 35 were selected (in 7 of them, this association was not adequately described or they were nonspecific population studies). The search platforms used were PubMed, Medline, Embase, Scopus, and virtual hospital libraries. All papers were considered regardless of language.

### 2.1. Statistical Analysis

A descriptive analysis of the categorical variables was carried out using absolute and relative frequencies, and in the case of numerical variables, analysis was done through the mean and standard deviation or median and the 25th and 75th percentiles, according to compliance with the assumption of normality. Bivariate analysis was carried out using Student's *t*-test or the Mann–Whitney *U* test to contrast numerical variables, and the chi-squared test or Fisher's exact test was used to contrast the hypotheses of categorical variables, as appropriate. The significance level was set at 0.05, and all tests were two-tailed. The statistical package SPSS version 24 was used.

## 3. Results

81 patients were included in the study here presented. Of these, 74 were those detected in the literature review [6–40] and 7 from our center. Of the total number of cases, 58 were men (71%), with a mean age of 53 years (±14.83), and a mean age of the onset of the disease was 34 years (±14.87). RA was the first disease diagnosed in 52% of cases. The mean duration of the disease (RA or AS) up to the time of diagnosis was 18 years (±13.16). The first symptom suffered was lower back pain in 48% (38/76), followed by arthritis in 46% (35/76) of the cases. 52% of the patients (38/73) had rheumatoid nodules, and 83% had suffered from inflammatory lower back pain at some point during their evaluation. Uveitis was detected in 13% of the patients (11/81). Extra-articular symptoms manifested in 18% (15/81), including 4 cases of Felty syndrome, most of which having manifested when conventional immunosuppressive therapy and/or biological therapy had not been available (Table 1).

Regarding imaging studies, spinal syndesmophytes were present in 73% of cases. 85% (65/76) presented erosions of the hands and/or feet in their radiographs. Radiological sacroiliitis was observed in almost all patients (80/81).

Regarding laboratory findings, RF was positive in 92% and, in 16 of 18 cases in which anti-CCP antibodies were quantified, these turned out to be positive. Finally, almost 90% of the patients were HLA B-27 positive (Table 2). No difference was of statistical significance because the sample size was small.

The treatment used was only carried out in 47 of the 81 cases. Of these, 83% had received a course of corticosteroids at one point in time. Nonsteroidal anti-inflammatory drugs were used in 59% of the cases. 25% of the patients were administered gold salts (with the last case being registered in 1995). Both hydroxychloroquine and sulfasalazine were used in 19% of the cases. With regard to methotrexate, there is evidence that the first patient was treated in 1993, with a total of 15 cases being treated. Anti-TNF-alpha therapy was used in 5 cases, in 2 of them infliximab and in another 2 adalimumab. Other less frequently used therapies were leflunomide, rituximab, D-penicillamine, azathioprine, and synoviorthesis (Table 3).

## 4. Discussion

In 1958, Wilkinson and Bywaters published a descriptive study of 222 patients with spondylitis that was carried out from 1940 to 1955 [36]. In one of the cases, a 55-year-old male patient was described as having symmetric arthritis, small joints, and subcutaneous nodules and was RF+. Later, in the evolution of his disease, he manifested typical AS symptoms, which at that time were diagnosed as “rheumatoid spondylitis.” This appears to be the first well-documented case of the coexistence of RA and AS. Based on this description, approximately 81 cases have been published (including 7 cases from our center). It is a highly infrequent association in a single patient that is a diagnostic challenge due to the similarities that exist between both diseases, mainly from a clinical perspective [4]. It is likely that the coexistence of both processes in a single patient is underestimated, as shown in a study in 2017, where in 286,601 patients with RA, the association with other autoimmune processes was evaluated, observing that 1.16% of patients also manifested AS [1].

AS and RA are considered to be two independent diseases due to their clinical, genetic, pathogenic, and analytical differences [3]. That is why the probability of the coexistence of these two entities in the same patient is low. However, some authors suggest that the initial cause that acts as a trigger in both diseases may be the same [28].

There are some different characteristics between each of these pathologies. For example, AS mainly affects the axial skeleton, and almost all patients report lower back pain with inflammatory characteristics, also associated with enthesitis. These signs and symptoms are usually asymmetric, rarely involving the upper limbs [28]. Regarding patients with RA, joint involvement is fundamentally symmetrical, predominantly in the upper limbs and rarely affecting the sacroiliac and iliolumbar areas [2].

From an analytical point of view, in RA, both the RF and anti-CCP antibodies are usually positive and of high value (approximately 70–80%), while in AS, only 10–15% are positive and of lower value [3, 4]. Regarding HLA B-27, its role throughout the course of the disease is not clear in patients with RA and it can be positive in up to 9% of cases [31].

In addition, imaging studies provide findings that are more characteristic of one disease than the other. For example, in RA, the erosive pattern is frequent, mainly affecting the proximal interphalangeal and metacarpophalangeal joints, with a symmetrical distribution, while in AS, these findings are exceptional, classically presenting an asymmetric and nonerosive pattern. Finally, the discovery of radiographic sacroiliitis is uncommon in patients with RA [31].

In this review, the ratio of males to females was approximately 3 to 1. One explanation for this could be that in almost half of the patients, the initial disease was AS, an entity that, as is known, mainly affects men (1.34). In patients with arthritis, this was mainly symmetric and it displayed an erosive pattern (85%).

56% of patients manifested subcutaneous nodules, a figure higher than that of patients with RA and without AS, which is approximately 40% [2]. 13% of patients presented uveitis throughout their disease; this figure is usually 20–30% in patients with AS and without associated RA [4].

Another important finding to highlight was the presence of syndesmophytes, found in 74% of cases after radiological studies. These last data are in accordance with the fact that the manifestation of AS plays a determining role in axial affectation when both entities coexist. 92% of the patients were RF+, and 90% were HLA B-27+. The previous statistic highlights the susceptibility of the patients' joints and puts into question the extent to which inflammatory rheumatic diseases have well-defined pathophysiological borders.

With regard to treatment, a lot of discrepancies still exist due to the emergence of new therapies such as disease-modifying antirheumatic drugs (DMARDs) and biological agents. In this sense, it should be noted that 25% of the registered cases were treated with gold salts (a therapy currently in disuse in most countries). With the data presented, we do not know whether the association of both diseases conditions a resistance to the treatments currently available for both RA and AS.

With these data and as a summary, in young patients diagnosed with RA who present with extra-articular manifestations (such as uveitis) and clinical activity data in the axial skeleton, the coexistence of ankylosing spondylitis should be suspected and further studies should be carried out (mainly HLA B-27, X-rays of the dorsolumbar spine/sacroiliac joints, and, where appropriate, MRI scans). Similarly, in patients with AS who manifest symmetric arthritis, mainly in the upper limbs, and with erosive lesions found in radiological studies, the possibility of a concomitant diagnosis of RA should be considered.

Regarding this work's weaknesses, the number of patients is limited, much of the published data is not very precise, and, in addition, the heterogeneity of the patients and the anachronism of the data make them difficult to analyze and, therefore, draw more precise conclusions.

Even so, to date, no other review exists that has been carried out in such an exhaustive manner. For this reason, we consider that its dissemination is important since it is likely that this association is being underestimated in clinical practice.

## 5. Conclusions

The association of RA and AS is highly infrequent; approximately 81 cases have been documented to date. The data presented in this review suggest that this association confers different characteristics since, in most cases, the patients have an erosive radiological pattern, RF and anti-CCP antibody positivity, and involvement of the axial skeleton and more frequently exhibit cutaneous nodules relative to patients with an isolated diagnosis of one of the two entities. More research is needed to support this conclusion.

## Figures and Tables

**Table 1:** Literature review on coexistence of rheumatoid arthritis and ankylosing spondylitis.

Author	No.	Sex	Age (years)	1st symptom	Onset (years)	1st disease	Duration (years)	Nodules	RF	Ac. CCP	HLA B-27	Lumbar pain	SI	Sind	Eros	Uveitis	Extra-art involv
Wilkinson and Bywaters	1	M	55	Arthritis	22	RA	33	+	+	NA	NA	+	+	NS	+	+	0
Martel and Duff	2	M	47	Arthritis	18	RA	29	0	+	NA	NA	+	+	0	+	0	0
	3	F	32	Arthritis	30	RA	2	+	+	NA	NA	+	+	+	+	0	0
	4	F	27	Arthritis	15	RA	12	+	+	NA	NA	0	+	+	+	0	0
	5	F	42	Arthritis	24	RA	18	+	+	NA	NA	0	+	NS	+	0	+
	6	M	52	Arthritis	21	RA	31	+	+	NA	NA	0	+	+	+	0	+
	7	M	37	Arthritis	29	RA	8	0	0	NA	NA	+	+	NS	+	+	+
	8	M	54	Arthritis	43	RA	9	+	+	NA	NA	+	0	NS	+	0	0
London and Bland	9	M	26	Arthritis	14	RA	12	+	NS	NA	NA	+	+	NS	NS	0	0
Rosenthal et al. [8]	10	M	73	Uveitis	22	AE	51	+	+	NA	NA	+	+	+	NS	+	+
Husskisson et al. [37]	11	M	80	L. pain	25	AE	55	+	+	NA	NA	+	+	+	+	+	0
Valkenburgh et al. [9]	12	F	45	Arthritis	36	RA	9	+	+	NA	NA	+	+	NS	+	+	+
Luthrah et al. [10]	13	M	59	L. pain	34	AE	25	+	+	NA	+	+	+	+	+	0	0
	14	M	65	L. pain	48	AE	17	+	+	NA	+	+	+	+	+	0	0
Querol et al. [11]	15	M	40	L. pain	18	AE	22	0	0	NA	+	+	+	0	0	0	0
	16	F	46	L. pain	27	AE	19	0	+	NA	+	+	+	0	+	0	0
	17	M	51	L. pain	25	AE	26	+	0	NA	+	+	+	+	0	0	0
	18	M	50	L. pain	42	AE	8	0	0	NA	+	+	+	+	0	0	0
	19	M	53	L. pain	41	AE	12	+	+	NA	+	+	+	+	+	0	0
	20	M	17	L. pain	15	AE	2	0	+	NA	+	0	+	0	+	0	0
	21	M	28	L. pain	15	AE	13	0	0	NA	+	+	+	0	0	0	0
Fallet et al. [35]	22	M	64	L. pain	30	AE	34	+	+	NA	+	+	+	+	+	0	0
	23	M	68	Arthritis	29	RA	39	0	+	NA	+	NS	+	NS	+	+	0
	24	F	68	Arthritis	65	RA	3	+	+	NA	+	0	+	NS	0	0	0
	25	M	54	Arthritis	40	RA	14	+	+	NA	+	+	+	NS	+	0	0
	26	M	67	Arthritis	57	RA	10	0	+	NA	0	+	+	+	+	0	0
	27	F	75	Arthritis	63	RA	12	0	+	NA	+	+	+	+	+	0	0
	28	F	58	Uveitis	42	RA	16	0	+	NA	+	0	+	NS	+	+	0
	29	M	68	L. pain	29	AE	39	0	+	NA	+	+	+	NS	+	0	0
	30	M	44	Myalgias	36	AE	8	+	+	NA	+	+	+	NS	+	0	0
Good et al. [12]	31	M	NS	L. pain	29	AE	NS	0	+	NA	+	+	+	+	+	0	0
	32	M	NS	L. pain	20	AE	NS	0	+	NA	+	+	+	+	+	0	0
	33	M	NS	L. pain	34	AE	NS	0	+	NA	+	+	+	0	+	0	0
Clayman and Reinertsen	34	M	48	L. pain	21	AE	27	+	+	NA	+	+	+	+	0	0	0
Espinoza et al. [14]	35	M	53	L. pain	47	AE	6	+	+	NA	+	+	+	NS	+	0	0
Major et al. [15]	36	M	64	L. pain	28	AE	36	+	+	NA	+	+	+	+	+	0	0
	37	M	61	L. pain	NS	AE	NS	0	+	NA	+	+	+	+	+	0	0
Lemmer and Irby	38	M	51	L. pain	26	AE	25	+	+	NA	+	+	+	+	+	0	+
Alexander et al. [17]	39	M	55	Arthritis	32	RA	23	+	+	NA	+	+	+	NS	+	0	+
	40	M	61	Arthritis	54	RA	7	+	+	NA	+	0	+	+	+	0	+
	41	M	NS	NS	NS	NS	NS	+	+	NA	0	NS	+	NS	0	0	0
	42	M	NS	NS	NS	NS	NS	+	+	NA	0	NS	+	NS	+	0	0
	43	M	NS	NS	NS	NS	NS	+	+	NA	+	NS	+	NS	+	0	0
Lavery et al. [18]	44	M	60	L. pain	18	AE	42	+	+	NA	+	+	+	NS	+	+	+
Alarcón Segovia and Martínez-Cordero	45	M	70	L. pain	NS	NS	NS	+	+	NA	+	+	+	+	+	0	0
Serrano et al. [20]	46	M	35	NS	22	RA	13	NS	+	NA	+	+	+	NS	NS	0	+
Fallet et al. [21]	47	M	52	Arthritis	25	RA	27	0	+	NA	+	+	+	+	+	+	+
	48	M	60	L. pain	48	AE	12	+	+	NA	+	+	+	+	+	0	0
	49	M	61	Arthritis	40	NS	11	0	+	NA	+	0	+	0	+	0	0
	50	M	56	L. pain	22	AE	34	0	+	NA	+	+	+	+	+	+	+
	51	M	66	Arthritis	61	RA	5	+	+	NA	+	+	+	0	+	0	0
	52	M	71	L. pain	21	AE	50	+	+	NA	+	+	+	+	+	0	0
	53	M	49	Arthritis	35	RA	14	0	+	NA	+	+	+	0	+	0	0
	54	F	83	Arthritis	69	RA	14	0	+	NA	+	+	+	+	+	0	0
Helfgott et al. [22]	55	M	77	L. pain	39	AE	38	+	+	NA	+	+	+	+	+	0	0
Sattar et al. [23]	56	M	35	L. pain	25	AE	10	0	+	NA	0	+	+	+	+	+	+
Martínez-Cordero et al. [24]	57	M	38	Arthritis	28	RA	10	NS	+	NA	+	+	+	+	+	0	0
Ferreiro Seoane et al. [25]	58	M	44	L. pain	31	AE	13	+	+	NA	+	+	+	NS	+	0	+
Toussirot and Acquaviva	59	M	48	Arthritis	22	RA	26	+	+	NA	0	0	+	0	+	0	0
	60	M	77	Arthritis	35	RA	42	0	+	NA	0	+	+	+	+	0	0
	61	F	27	Arthritis	19	RA	8	0	+	NA	+	+	+	0	+	0	0
Genc et al. [27]	62	F	62	Arthritis	52	RA	10	NS	0	NA	+	+	+	+	+	0	+
Guo et al. [28]	63	F	30	L. pain	23	AE	7	0	+	+	+	+	+	NS	+	0	0
Baksay et al. [29]	64	F	57	L. pain	25	AE	32	NS	+	+	+	+	+	+	+	0	0
Feijo et al. (2011)	65	F	65	L. pain	64	AE	1	0	+	+	+	+	+	+	+	0	0
Aghdashi et al. [31]	66	F	70	Arthritis	58	RA	12	NS	+	+	+	0	+	+	+	0	0
Dundar et al. [32]	67	M	63	L. pain	51	RA	12	NS	+	+	NS	+	+	NS	+	0	0
Koca et al. [38]	68	F	38	L. pain	38	RA	1	0	+	+	0	+	+	0	0	0	0
Barczynska et al. [33]	69	F	55	Arthritis	35	RA	20	+	+	+	+	+	+	+	+	0	0
	70	M	56	L. pain	34	AE	22	NS	+	0	+	+	+	+	+	0	0
	71	M	65	NS	NS	NS	NS	NS	+	0	+	+	+	+	+	0	0
Baccouche et al. [34]	72	F	21	L. pain	20	AE	1	0	+	+	+	+	+	+	+	0	0
Sargin and Gurer	73	F	47	L. pain	45	RA	2	0	NS	NS	NS	+	+	NS	NS	0	0
Haridas and Kiran	74	F	32	Arthritis	32	AR	1	0	+	+	+	+	+	NS	NS	0	0
Flores-Robles et al. (2020)	75	M	55	L. pain	41	AE	14	0	+	+	+	+	+	+	0	0	0
	76	F	43	Arthritis	35	RA	8	0	+	+	+	+	+	0	+	0	0
	77	F	64	Arthritis	51	RA	13	0	+	+	+	0	+	0	0	0	0
	78	M	57	Arthritis	28	RA	29	+	+	+	+	0	+	0	+	0	0
	79	M	64	L. pain	49	AE	15	0	+	+	+	+	+	+	0	0	0
	80	M	63	Arthritis	47	RA	16	0	+	+	+	+	+	+	+	0	0
	81	F	75	Arthritis	70	RA	5	0	+	+	+	+	+	+	+	0	0

Ac. CCP: citrullinated peptide antibodies; AE: ankylosing spondylitis; Eros: radiographic erosions on hands and/or feet; extra-art involv: extra-articular involvement; F: female; L. pain: lumbar pain; M: male; NA: not applicable; NS: not specified by the author; RA: rheumatoid arthritis; RF: rheumatoid factor; SI: radiological sacroiliitis; Sind: spine syndesmophytes; sign +: existence/positive; sign 0: absence/negative.

**Table 2:** Clinical and epidemiological characteristics of 81 patients with concurrent RA and AE.

	*n* = 81
Sex	
Male	58 (71.60%)
Mean age (years)^*∗*^	53.72 ± 14.83
Onset mean age (years)^*∗*^	34.89 ± 14.47
First disease diagnosed	
Rheumatoid arthritis	39/75 (52%)
Ankylosing spondylitis	36/75 (40%)
Mean duration of disease (years)^*∗*^	18.08 ± 13.16
First symptom	
Lumbar pain	37/76 (48.68%)
Arthritis	35/76 (46.05%)
Uveitis	2/76 (2.81%)
Rheumatoid nodules	38/73 (52.05%)
Inflammatory lumbar pain	64/77 (83.12%)
Uveitis	11/81 (13.58%)
Extra-articular involvement (except uveitis)	15/81 (18.51%)
Felty syndrome	4/16
Reactive arthritis	3/16
Sjögren's syndrome	2/16
Vasculitis	2/16
Membranous nephropathy	2/16
Dermatomyositis	1/16
Interstitial lung disease	1/16

^*∗*^Mean ± standard deviation.

**Table 3:** Radiological, laboratory, and treatment characteristics.

	*n* = 81 (percentage)
Syndesmophytes (radiograph/MR/CT)	42/57 (73.68%)
Radiographic erosions	65/76 (85.52%)
Radiological sacroiliitis	80/81 (98.76%)
Positive RF	73/79 (92.40%)
Citrullinated peptide antibodies	16/18 (88.88%)
Positive HLA B-27	60/67 (89.55%)
Treatment	
NSAIDs	28/47 (59.57%)
Corticosteroids	39/47 (82.97%)
Gold salts	12/47 (25.53%)
Hydroxychloroquine	9/47 (19.14%)
Sulfasalazine	9/47 (19.14%)
Methotrexate	15/47 (31.91%)
Leflunomide	4/47 (8.51%)
TNF-*α*-i	5/47 (10.63%)
Others	6/47 (12.76%)

CT: computerized tomography; MR: magnetic resonance, NSAIDs: nonsteroidal anti-inflammatory drugs; RF: rheumatoid factor; TNF-i: tumor necrosis factor inhibitor.
